# Data Resource Profile: Australia’s Person Level Integrated Data Asset (PLIDA)

**DOI:** 10.1093/ije/dyag202

**Published:** 2026-09-14

**Authors:** Carrie Samuels, Rosemary J Korda, Sergey Timonin, Phillip Gould, Jennifer Welsh

**Affiliations:** National Centre for Epidemiology and Population Health, The Australian National University, Canberra, ACT, Australia; Data Services, Infrastructure and Methods Group; Australian Bureau of Statistics, Canberra, ACT, Australia; National Centre for Epidemiology and Population Health, The Australian National University, Canberra, ACT, Australia; National Centre for Epidemiology and Population Health, The Australian National University, Canberra, ACT, Australia; Data Services, Infrastructure and Methods Group; Australian Bureau of Statistics, Canberra, ACT, Australia; National Centre for Epidemiology and Population Health, The Australian National University, Canberra, ACT, Australia

**Keywords:** linked data, administrative data, whole population data, census, record linkage, Australia

Key Features

- Australia’s Person Level Integrated Data Asset (PLIDA) was established in 2015 by the Australian Bureau of Statistics (ABS) to realize the research and policy value of Australian government data.
- PLIDA contains linked national administrative and survey datasets relating to health, disability, education, social services, income, taxation, employment, migration, civil registration, and demographics. This breadth of national Australian data is a unique feature of PLIDA.
- Datasets are added to PLIDA by linkage to a spine, designed to contain a record for each person resident in Australia at any point from 2006 onwards. The spine is constructed through linkage of three administrative datasets associated with universal health insurance, social security, and taxation, resulting in near-complete longitudinal coverage of this population. The spine included around 40 million records as of June 2025.
- Datasets are updated on a rolling basis with a mix of sub-annual, annual, and less frequent updates. New datasets are added in response to identified research and policy needs.
- PLIDA data are available to Australian and international researchers. Access is by application to the ABS and subject to approval by data custodians. Approved researchers access data through the ABS DataLab, a secure cloud environment.

## Data resource basics

The Person Level Integrated Data Asset (PLIDA) is Australian whole-of-population linked data infrastructure containing person-level data with near-complete national population coverage. PLIDA contains administrative and survey datasets relating to several domains, including health, disability, education, social services, income and taxation, employment, migration, civil registration, and demographics. These linked data enable cross-domain analyses, generating insights and knowledge unattainable from unlinked data.

PLIDA was established by the Australian Bureau of Statistics (ABS) in partnership with other Australian government agencies to realize the research and policy value of Australian government data. Development commenced in 2015, and PLIDA (known as the Multi-Agency Data Integration Project [MADIP] data asset until 2023) has been available to Australian researchers since 2018 and international researchers since 2025.

**Figure 1:**
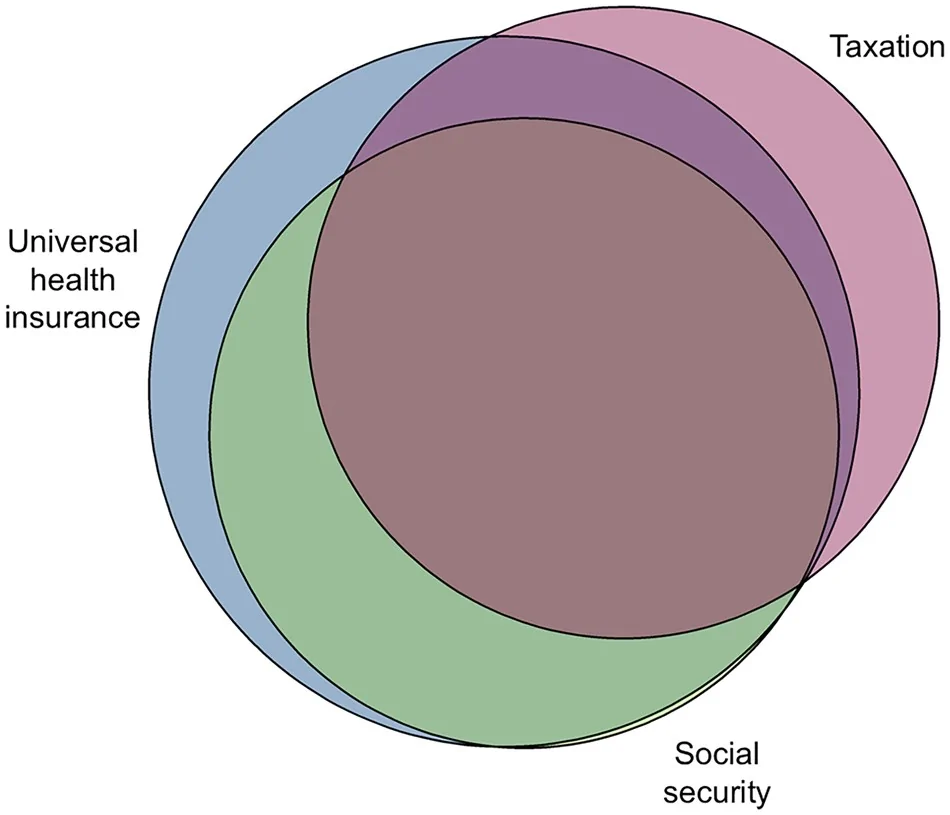
Spine entities by combination of contributing dataset records. The universal health insurance dataset is the Medicare Consumer Directory (MCD). The social security dataset is Data Over Multiple Individual Occurrences (DOMINO) Centrelink Administrative Data. The taxation dataset is Personal Income Tax (PIT) data. Area is proportional to size for the June 2025 spine, which had the following sized subsets: 19.35M spine entities with records from all three of MCD, DOMINO, and PIT, 7.99M spine entities with records from MCD and DOMINO, 4.77M spine entities with records from PIT only, 4.12M spine entities with records from MCD only, 3.49M spine entities with records from MCD and PIT, 0.11M spine entities with records from DOMINO only, and 0.04M spine entities with records from DOMINO and PIT (not illustrated due to geometric constraints). Adapted from the Australian Bureau of Statistics’ Person Linkage Spine June 2025 Methodology and Quality Assessment (unpublished, available to approved researchers)

PLIDA is built around a linkage spine [1], designed to include a single entity for each person resident in Australia at any point from 2006 onwards (hereafter, ‘ever-resident’). The spine is constructed by linking three administrative datasets, which in combination have near-complete coverage of the ever-resident population: Medicare (universal health insurance), Centrelink (social security), and taxation, collectively referred to as ‘the spine datasets’ (Table 1). This approach is necessitated by the absence of a national population register or similar dataset and was informed by the approach used for New Zealand’s Integrated Data Infrastructure [5]. The spine is rebuilt annually for a reference period ending 30 June to update existing data and add records for new residents. As the spine datasets do not share a common unique identifier, linkage is based on first name, surname, address, date of birth, sex or gender, and country of birth (hereafter, ‘linkage variables’). Probabilistic linkage methods have been used to build the spine since the 2025 update. Prior to this, deterministic methods involving a series of decision rules based on full or partial agreement between various combinations of the linkage variables were used [6–8]. A spine entity, which can include records from any combination of the three spine datasets, is created for each person and assigned a unique person identifier (spine ID; Fig. 1). The change in linkage method improved efficiency without affecting the quality, structure, or composition of the spine. Linkage occurs under the separation principle; the linkage process is isolated from the preparation of data for researcher access [9].

**Table 1:** Characteristics of the three datasets used to create the spine.

Characteristic	Medicare Consumer Directory (MCD)	Data Over Multiple Individual Occurrences	Personal Income Tax (PIT)
		(DOMINO) Centrelink Administrative Data	
Dataset coverage	People registered with Medicare, Australia’s universal healthinsurance program.	People registered with Centrelink, Australia’s social security program. This includes people who have applied for social security payments or concession cards, as well as their partners and children where these relationships are relevant to determining eligibility.	People registered with the Australian Tax Office (ATO).
Eligibility	The following people are eligible for Medicare [2]: Australian citizens and permanent residents residing in Australia. Australian citizens who have been absent from Australia for up to 5 years. New Zealand citizens residing in Australia. Residents of select countries that have Reciprocal Health Care Agreements with Australia may enrol for the duration of their Australian visa. Temporary visa holders are generally ineligible, except for those covered by a Reciprocal Health Care Agreement or a ministerial order.	The following people are eligible to apply for social security, subject to meeting other specific eligibility requirements (e.g. age, income, disability) [3]: Australian citizens and permanent residents residing in Australia, subject to waiting periods for newly arrived residents. Australian citizens and permanent residents living in select countries that have international social security (ISS) agreements with Australia (specific payments only). New Zealand citizens residing in Australia, subject to additional eligibility restrictions. People residing in Australia who are from countries that have ISS agreements with Australia (specific payments only). Temporary visa holders are generally ineligible, except for those covered by ISS agreements or a ministerial determination.	People who expect to pay income tax in Australia can maintain registration with the ATO, regardless of residency.^a^ [4] Registration is also required to apply for social security, with limited exceptions [3].
Spine inclusion criteria	Includes: all records that: Relate to a person who had an active Medicare entitlement at any point from 2006 onwards.	Includes: all records that: Relate to a person who received a social security payment or had an active concession card at any point from 2006 onwards, or Relate to a partner or a child of a person who met the above criteria, only if the partner or child record also links to an MCD or PIT record.	Includes: all records that: Relate to a person who submitted an individual tax return or received employee income at any point from 2006 onwards, or Relate to a person granted an Australian visa from 2006 onwards.^b^
	Excludes: Records with missing data for three or more linkage variables.^c^ Records where a single dataset identifier seemingly contains information for more than one unique person.	Excludes: Records with missing data for three or more linkage variables.	Excludes: Records with missing data for three or more linkage variables.
Number of records included in the June 2025 spine	34.95M	27.49M	27.65M

Information is current at the time of writing and is based on the June 2025 spine. The PIT dataset is built from the ATO Client Register (taxpayer registrations), supplemented by Income Tax Return datasets (individual tax returns), and Payment Summary and Single Touch Payroll datasets (employee income received), which are used to apply the spine inclusion criteria.

a While registration with the ATO is not compulsory, taxpayers who do not provide a registration number have tax withheld at the highest marginal rate, meaning that in practice, virtually all taxpayers are registered.

b Based on visa information from a separate dataset, which is linked to the ATO Client Register dataset via a shared unique migrant identifier.

c Linkage variables include first name, surname, address, date of birth, country of birth, and sex or gender.

All other datasets are added to PLIDA by linkage to the spine, either via a shared unique identifier (e.g. Medicare identifier) or with the same probabilistic linkage methods used to construct the spine (deterministic methods prior to 2026). This adds the spine ID to linked records, creating indirect links between all datasets and enabling them to be joined at the person level. All dataset records, regardless of whether they link to a spine entity, are included in PLIDA, enabling analysis of both linked and unlinked data, and of linkage outcomes.

The ABS undertakes ongoing research and development to improve the spine and linkage methods. Enhancements under consideration at the time of writing include increasing the frequency of spine updates, extending temporal coverage, and addressing population coverage gaps.

## Data collected

PLIDA contains datasets available for general use via a standard application process, as well as datasets linked only for a specific project. The former constitute the PLIDA modular product, which included 40 datasets at the time of writing (Table 2).

**Table 2:** Descriptions of the datasets and Core modules in the PLIDA modular product.

Name	Description	Reference period	Update periodicity	Lag in currency
**Core modules** ^a^				
Core Demographics	Demographic information (e.g. gender,^b^ month and year of birth) including derived ‘best’ values.	2006–	Yearly (FY)	12 months
Core Locations	Australian Statistical Geography Standard structures and de-identified address codes, including derived address histories.	2006–	Yearly (FY)	12 months
Core Scoping	Information relevant to defining a study population for a given time period, including data related to travel, migration, and death, as well as derived activity flags suitable for use with signs-of-life methods.	2006–	Yearly (FY)	12 months
Core Relationships	Parent–child and spousal relationship information including derived relationship histories.	2006–	Yearly (FY)	12 months
Core Income	Income received from various sources including employment, social security, and investments.	2006–	Yearly (FY)	12 months
**Health datasets**				
Medicare Consumer Directory	Demographic data for people registered for Medicare, Australia’s universal health insurance program.	2006–	Yearly (FY)	2 weeks
Medicare Benefits Schedule	Claims data for Medicare-funded services, including item number, date of service, fee charged, and benefit paid.	2006–	Quarterly	6 weeks
Pharmaceutical Benefits Scheme	Dispensing data for Medicare-funded pharmaceuticals, including item code, date of supply, quantity supplied, benefit amount, and patient contribution amount.	2006–	Quarterly	6 weeks
Australian Immunisation Register	Records of vaccinations administered in Australia, or overseas where recorded by a recognized Australian vaccination provider, including vaccination type and date administered.	2010–	Monthly	2 weeks
National Health Survey	Cross-sectional survey data from the ABS National Health Survey, including information on health behaviours, risk factors, and health conditions.	2014–2015 2017–2018 2020–2021	Follows survey schedule	Varies
National Study of Mental Health and Wellbeing	Cross-sectional survey data from the ABS National Study of Mental Health and Wellbeing, including information on mental health disorders and self-harm behaviours.	2020–2022	Follows survey schedule	Varies
Patient Experience Survey	Cross-sectional survey data from the ABS Patient Experience survey, including information on access to, barriers to, and experiences of healthcare services.	2023–2024	Follows survey schedule	Varies
**Disability and ageing datasets**				
National Disability Insurance Scheme	Participant and applicant data for the National Disability Insurance Scheme, an Australian social insurance program. Includes demographics, disability, funded supports with payment amounts, service providers, and participant outcomes.	2013–	Quarterly	6 weeks
National Aged Care Data Clearinghouse	Information about people receiving government-funded aged care, including residential care, home care, and home support. Includes demographics, periods of care, and types and dates of support services.	2015–	Quarterly	6 months
Survey of Disability, Ageing, and Carers	Cross-sectional survey data from the ABS Survey of Disability, Ageing, and Carers, including information on disability status, long-term health conditions, need for assistance, informal care, and living arrangements.	2018, 2022	Follows survey schedule	Varies
**Population and migration datasets**				
Census of Population and Housing	Cross-sectional^c^ data from the ABS Census of Population and Housing, including information on demographics, cultural background, education, and employment.	2011, 2016, 2021	5-yearly	Varies
Australian Census Longitudinal Dataset	A 5% sample of longitudinally linked Census records.	2006–2021	5-yearly	Varies
Death Registrations	Information about people whose death is registered in Australia, including demographics and date and cause of death.	2007–	Yearly (CY)	12 months
Birth Registrations	Demographic information about people whose birth is registered in Australia, and their parents.	2006–	Yearly (CY)	12 months
Migrant and Traveller Demographics	Demographic and location information about people who have migrated or travelled to or from Australia	1990–	Yearly (CY)	8 months
Visa Applications and Grants	Information about people who have applied for a visa to live in Australia, including visa grant date, subclass, and stay period.	1990–	Yearly (CY)	8 months
Settlements Database	Information about people who have migrated permanently to Australia, including arrival date, occupation, and foreign citizenship	2000–	Yearly (CY)	8 months
Travellers	Information about people who have travelled in or out of Australia, including flags for residence and physical presence in Australia	2006–	Quarterly	2 months
Adult Migrant English Program	Information about participants in the Adult Migrant English Program, a free service for eligible migrants to improve their English language skills.	2003–2019	Not scheduled	N/A
**Social services datasets**				
Data Over Multiple Individual Occurrences (DOMINO) Centrelink Administrative Data	Benefit data for recipients of Centrelink, Australia’s social security program, including benefit type, start and end dates, and amounts paid.	2000–	Quarterly	6 weeks
Data Exchange	Information about clients of select programs funded by government grants, reported through the Data Exchange, a Department of Social Services reporting tool. Includes demographics, session attendance, and client outcome assessments.	2015–	Yearly (CY)	2 months
**Education datasets**				
Higher Education	Information about students studying undergraduate and postgraduate qualifications at Australian institutions, including demographics, field of education and course dates.	2005–2021	Not scheduled	N/A
Total Vocational Education and Training Activity	Information about students studying nationally recognized training at Australian vocational education institutions, including demographics, course enrolment, and course completion.	2015–	Yearly (CY)	10 months
Apprentice and Trainee	Information about Australian apprentices supported by Commonwealth programs, including demographics, qualifications, periods of employment, and Commonwealth payments received.	2006–	Yearly (CY)	12 months
Australian Early Development Census	Cross-sectional data collected in the Australian Early Development Census, a national collection of early childhood development data for children in their first year of school, collected by their teachers.	2009, 2012, 2015, 2018, 2021, 2024	3-yearly	Varies
**Income, taxation, and employment datasets**				
Labor Force Survey	Rotating panel survey data from the ABS Labour Force Survey, including information on employment, unemployment, labour force participation, and hours worked.	2023–	Monthly	1 month
Australian Public Service Employment Database	Employment records for current and former Australian Public Service employees, including demographics, employment level, and standard hours worked.	2005–2024	Not scheduled	N/A
Australian Tax Office Client Register	Demographic information about people who have registered with the Australian Tax Office, which is required to interact with the Australian tax and welfare systems.	1999–	Yearly (FY)	8 weeks
Income Tax Returns	Information from individual annual personal income tax returns submitted to the Australian Tax Office, including income, losses, deductions, expenses, offsets, tax withheld, and debts.	1999–	Yearly (FY)	15 months
Single Touch Payroll	Information about payments made to employees of Australian businesses, reported by employers at time of payment through payroll software. Includes gross payment amounts and reportable employer superannuation contributions.	2020–	Monthly	4 months
Payment Summaries	Information about payments made to employees of Australian businesses, reported by employers as an annual summary. Includes gross payments, tax withheld, and reportable employer superannuation contributions.	2001–	Yearly (FY)	8 months
Business Ownership	Concordances between people and the businesses they own, which can be used to link with the Business Longitudinal Analysis Data Environment (BLADE), the ABS business-level linked data infrastructure.	2009–	Yearly (FY)	15 months
Superannuation Accounts Extract	Information about money held by individuals in superannuation, Australia’s compulsory retirement savings scheme. Includes account balances, employer contributions, and personal and other contributions.	2018–	Yearly (FY)	15 months
Member Contributions Statements	Information about money held by individuals in superannuation, including account balances, employer contributions, and personal and other contributions.	1999–2018	Ceased	N/A
Self-Managed Superannuation Funds	Information about money held by individuals in self-managed superannuation funds, including account balances, employer contributions, and personal and other contributions.	2007–	Yearly (FY)	24 months
Capital Gains Tax	Information about individuals or sole traders’ capital gains and losses from the disposal of assets such as property and shares.	2000–	Yearly (FY)	24 months
Rental Property Schedule	Information about rental property income and expenses for individuals, trusts and partnerships.	1999–	Yearly (FY)	12 months
COVID-19 Early Release of Superannuation scheme	Information about individuals’ requests for early access to their superannuation under the COVID-19 Early Release of Super scheme.	2020	Ceased	N/A
JobKeeper Payment	Information about payments to eligible employees under the JobKeeper Payment scheme, a COVID-19 employment support program.	2020–2021	Ceased	N/A
JobMaker Hiring Credit scheme	Information about payments made for eligible employees under the JobMaker Hiring Credit Scheme, a COVID-19 employment support program.	2020–2023	Ceased	N/A

Information is current at the time of writing and is adapted from the March 2026 PLIDA modular product data item list. Refer to the ABS website for an up-to-date list [10].

Datasets that are updated yearly can be updated on either a calendar year (CY) or financial year (FY, 1 July–30 June) basis. Lag in currency refers to the lag between the end of the updated dataset reference period and the time when the dataset becomes available to researchers. Where update periodicity is ‘not scheduled’, datasets may be updated in the future, but timing is uncertain. Where update periodicity is ‘ceased’, datasets will not be further updated.

a The Core modules combine related variables from several datasets, including the spine datasets and the Census, and include derived ‘best’ values. In most cases, the source data are also included to enable alternative derivations.

b The derived ‘gender’ variable in the Core Demographics module is constructed from undifferentiated sex and gender data.

c Restrictions apply to longitudinal analysis of Census data. Researchers can access either a single cross-sectional Census dataset, or the 5% sample of longitudinally linked Census data. These can be joined with non-Census datasets, but access controls prevent the joining of two or more full Census datasets with each other.

ABS: Australian Bureau of Statistics.

The PLIDA modular product contains predominantly federal government administrative data but also includes the Census of Population and Housing (hereafter, Census) and other nationally representative survey data collected by government agencies (e.g. the ABS National Health Survey). Selected state and territory administrative datasets, including birth and death registrations, are also incorporated through national collections.

Each dataset in the PLIDA modular product is structured as one or more modules, and access is provided only to the modules required for each project. Where datasets are split into modules, this is done by grouping variables thematically or as otherwise required by data custodians (government agencies or other organizations responsible for datasets). Datasets are updated on a rolling basis with a mix of sub-annual, annual, and less frequent updates. New datasets are added in response to identified research and policy needs, subject to availability of funding and approvals by data custodians.

Datasets can also be linked with PLIDA for project-specific use without being included in the PLIDA modular product. This occurs when data custodians do not approve broader access; such additions must be funded by the requesting project.

The PLIDA modular product also includes ‘Core’ modules curated by the ABS (Table 2). These thematically collate key variables from the spine and other selected datasets for all spine entities. The Core modules also provide derived variables expected to be of broad use. For example, the Core Demographics module includes derived month and year of birth, gender, country of birth, and date of death. These are derived from selected datasets using rules-based methods to resolve conflicts (e.g. most frequent values of month and year of birth). The source variables from contributing datasets are also included, enabling use of alternative derivations.

Disclosure risk for PLIDA data is managed through the Five Safes framework, including treatment of direct identifiers prior to researcher access [11, 12]. Names are removed, and dates of birth are reduced to month and year. Addresses are coded to both Australian Statistical Geography Standard [13] structures and de-identified address keys. Free-text variables are removed because of the risk they may contain identifying information.

## Data resource use

PLIDA supports a broad range of analyses, from descriptive through to predictive and causal. While PLIDA has been used by researchers from a range of disciplines [14], the following examples focus on epidemiological applications.

PLIDA is well suited for descriptive research, including routine government reporting, particularly as it allows for disaggregated analyses across subpopulations not commonly identifiable in unlinked administrative datasets. Examples include routine reporting of COVID-19 vaccination uptake among culturally and linguistically diverse populations [15], studies quantifying socio-economic inequalities in mortality [16], vaccination uptake [17], and out-of-pocket healthcare costs [18], and studies describing risk factors associated with suicide [19], younger onset dementia [20], and psychotropic medication use in older adults [21]. Analysis of unlinked data is also possible. For example, one study estimated treatment persistence in rheumatoid arthritis from unlinked pharmaceutical claims data [22].

PLIDA has also been used for predictive and causal analyses, notwithstanding the challenges of drawing causal inferences from observational data [23]. Examples include using Pharmaceutical Benefits Scheme (PBS) data to construct the Rx-Risk Index, a validated predictor of overall health [24]; estimating the real-world vaccine effectiveness of COVID-19 vaccination against COVID-19 specific and all-cause mortality in older Australians [25]; evaluating the impact of eligibility reassessment of young recipients of disability support payments on healthcare use [26]; and assessing the effects of increased financial incentives for rural general practitioners on provision of care with zero patient out-of-pocket costs [27].

## Strengths and weaknesses

As linked data infrastructure, many of PLIDA’s strengths and limitations are inherent in the types of data it contains. The benefits and challenges of working with administrative, survey, and linked data are well documented [28–34]; understanding how these manifest in PLIDA is essential for appropriate analysis.

The inclusion of both administrative and survey data in PLIDA allows researchers to capitalize on the strengths of each data type. Administrative data are generally longitudinal and have complete coverage of the population interacting with the relevant administrative program, enabling research on small subpopulations. However, they can lack detailed demographic and other contextual information not directly relevant to program operation. In contrast, survey data often include detailed self-reported information on a wide range of topics, including those not typically recorded in administrative sources. However, these data are collected less frequently (and often cross-sectionally), subject to self-reporting errors, and are, except for censuses, limited to relatively small samples of the population. Linkage of these data broadens research opportunities, enabling administrative data to enrich survey findings or add longitudinal follow-up of survey respondents, and survey data to supplement administrative records.

A key strength of PLIDA is the breadth of information available across multiple domains. In addition to health data, PLIDA incorporates data about education, migration, income, taxation, and employment, enabling analysis of associations between health outcomes and factors operating outside of the health system (e.g. social determinants of health). Further economic data are available in the Business Longitudinal Analysis Data Environment (BLADE) [35], ABS-managed business-level linked data infrastructure that can be joined with PLIDA using known business-person relationships, such as employer-employee relationships recorded in taxation data. This breadth of data, the inclusion of data related to the delivery of government programs, as well as the ability to add new data in response to emerging demands positions PLIDA as highly suited for policy-relevant research, including evaluation of government policy.

Although linkage rates are typically high, linkage error (both missed and false links) is present in PLIDA and can introduce bias. Linkage rates are nearly 100% for the Medicare Benefits Schedule (MBS) and PBS datasets (which share a common identifier with the spine), above 95% for death registrations and immunization records, and above 90% for most survey datasets. However, linkage rates are lower for datasets that have substantial missing data for linkage variables or contain relatively larger proportions of records for persons not included in the spine (e.g. migration datasets). While the ABS produces linkage reports for each dataset, researchers should quantify and consider linkage rates as they relate to their study, noting these will be study-specific due to selection of a study population and compounding of linkage errors when multiple datasets are brought together via the spine [1, 16]. Understanding and assessing the impact of linkage error in PLIDA can be challenging. The implications of linkage error depend on whether the dataset is being used to define the study population (where it can contribute to selection bias), or measure exposures or outcomes (where it can contribute to information bias), and the extent to which the error is differential, which may result in bias even when linkage rates are high overall [33].

A key challenge when using PLIDA is selecting appropriate study populations, particularly when the target population is not fully covered by a single dataset or the Core modules. These, along with other datasets with near-complete longitudinal or cross-sectional population coverage, still have both under- and over-coverage of the ever-resident population (Table 3). This reflects factors such as the ineligibility of temporary visa holders for specific government programs, the inclusion of visitors to Australia in some datasets, and data quality. Population under- and over-coverage may be meaningful limitations for some studies. The Core Scoping module contains data that can be used to define study populations, including travel, migration, and death data that enable exclusion of records for persons who were deceased or not resident during a study period. However, these data are incomplete prior to 2007, and subject to linkage error, leading to incomplete exclusion of records, and thus additional over-coverage. To mitigate this, the Core Scoping module includes activity flags that can be used to reduce over-coverage using the ‘signs-of-life’ method, where activities recorded in administrative data are used to infer presence in the population [37].

**Table 3:** Coverage of the Core modules and other PLIDA datasets with near complete coverage of the resident population of Australia.

Characteristic	Core modules	Medicare Consumer Directory (MCD)	Census of Population and Housing (Census)
Dataset coverage	People with a spine entity, being those who met at least one of the following criteria at any point from 2006 onwards: Had an active Medicare entitlement. Received a social security payment or had an active concession card. Submitted a personal tax return, received employee income, or had a migration record linked to their tax record.	People with an active Medicare entitlement at any point from 2006 onwards.	People who were recorded in the Census [36].
Temporal coverage	Longitudinal	Longitudinal	Cross-sectional
Under-coverage of the ever-resident population (2006–)	Excludes most non-working temporary visa holders, except for a small number who are eligible for Medicare or social services.	Excludes most temporary visa holders, except for a small number who are eligible for Medicare.	Excludes people temporarily overseas on Census night, those who migrated from Australia prior to Census night, and those who migrated to Australia after Census night. Excludes people who had died before or were born after Census night. Excludes people who were in Australia on Census night but did not respond to the Census, except in cases where records are imputed for them. Further under-coverage will arise if unlinked Census records, which include all imputed records, are excluded.
Over-coverage of the ever-resident population (2006–)	Includes some people seemingly not ever-resident from 2006, evidenced by the presence of records with dates of death prior to this period. Includes visitors to Australia who enrolled in Medicare under a reciprocal healthcare agreement. May include people who were not ever-resident from 2006 but retained eligibility for Medicare or Centrelink, or paid income tax in Australia during any of this period. May include unidentified duplicates, where spine dataset records belong to the same person but were not linked.	Includes some people seemingly not ever-resident from 2006, evidenced by the presence of records with dates of death prior to this period. Includes visitors to Australia who enrolled in Medicare under a reciprocal healthcare agreement. May include people who were not ever-resident from 2006 but retained eligibility for Medicare during any of this period.	Includes people visiting Australia on Census night. Includes some duplicate person records, where a person was enumerated multiple times.

Collectively, the Core modules include data for all spine entities, though individual modules vary in coverage. Refer also to the detailed spine inclusion criteria and Medicare eligibility criteria (Table 1). The Census is conducted every 5 years. Data from the Censuses for 9 August 2011, 9 August 2016, and 10 August 2021 are available in PLIDA at the time of writing. Future Census data are expected to be added to PLIDA soon after their release. The Census aims to count every person in Australia on Census night, including visitors to Australia, but excluding people in Australian external territories, foreign diplomats and their families, and foreign crew members on ships. In practice, there is some non-response; the person-level response rates were 96.3%, 94.8%, and 95.8% in 2011, 2016, and 2021. Records are imputed for people in private dwellings for which no Census form was returned, and for people in non-private dwellings who do not return a Census form, but not in cases where one or more people in an otherwise responding private dwelling do not respond. Imputed records cannot be linked to the spine but are still included in PLIDA.

Sparse information on health indicators as well as protective and risk factors is another challenge when using PLIDA. Such information is often required to measure health outcomes, account for healthcare need, and control for confounding. While detailed health measures (e.g. psychological distress, body mass index, and smoking status) are included in some survey datasets in PLIDA, they are only available for cross-sectional samples of the population. The MBS dataset includes information on consultations with health practitioners, pathology tests, and diagnostic imaging services. However, reason for visit, test results, and diagnoses are not recorded. Hospital data containing diagnostic and procedure codes, as well as many other health data routinely collected by states (e.g. cancer registries) are not currently available in the PLIDA modular product. However, some of these data have been linked with PLIDA for specific projects and may be added to the PLIDA modular product in the future.

Many administrative datasets in PLIDA relate to highly complex government programs and are challenging to use for research purposes. These programs have differing eligibility criteria, rules and procedures, and data capture methods, which may change over time, often with critical implications for research. Failure to understand and account for these complexities can result in inappropriate use of the data and erroneous conclusions. Examples relevant to PLIDA include collection of undifferentiated sex and gender data [38]; under-recording of pathology tests in MBS data [39]; and absence of PBS dispensing records for medicines not listed on the PBS, paid for privately, supplied over the counter, or (prior to July 2012) costing less than the co-payment threshold [40]. Timing of events recorded in administrative data may not align with the true timing of exposures, outcomes, or changes to demographic or contextual characteristics. Researchers must consider this when defining cohort entry, exposure timing, and follow-up periods.

To support appropriate use of PLIDA, the ABS provides a user guide, spine methodology report, linkage reports, and explanatory notes to approved researchers. However, researchers should still plan to consult with people with expertise on the various administrative programs and invest substantial effort into understanding limitations of the data. To facilitate knowledge sharing and efficient research, there is a growing PLIDA community of practice, including working groups and a shared code library, to which all researchers can contribute.

## Data resource access

Researchers apply to access PLIDA through an online portal. Access is charged on a cost-recovery basis. Supporting information, including the PLIDA data item list, current charges, and contact details, is available online [10, 41]. The ABS manages the application process, including approval of requested modules by the relevant data custodians. Modules are approved individually; timeframes vary with project complexity and custodian capacity. At the time of writing, data access is granted for most modules within 2 to 6 weeks of application. Projects must be for research purposes and of demonstrable public benefit. Projects materially concerned with the lives of Aboriginal and Torres Strait Islander or Māori and Pasifika people or communities in Australia are referred to an ABS Cultural Review Panel.

To apply, researchers must either be from academic or public policy institutions, be government employees, or be contractors or individuals from private sector organizations sponsored by government. Applications from researchers outside Australia are assessed on a case-by-case basis. Applicants must complete safe researcher training covering their responsibilities and the legislative requirements of data users.

PLIDA is accessed in the ABS DataLab, a trusted research environment administered by the ABS. Access is via remote desktop to a secure cloud environment without external connectivity. In DataLab, researchers can access PLIDA explanatory material and use analytical software including R, SAS, Stata, Python, and Databricks. Final analytical outputs must comply with output rules and are only egressed from the DataLab after checking by ABS officers. Researchers cannot discuss any results that have not been cleared through this process, except with other approved researchers on their project.

Publication protocols apply to research using PLIDA. Two weeks’ notice of publications must be provided to data custodians via the ABS, although custodians cannot veto publications. The ABS provides a preferred citation structure for PLIDA, as well as required standard acknowledgement wording for some datasets.

## Ethics approval

The ABS creates and manages PLIDA in accordance with its mandate to collect, compile, analyse, and disseminate statistics, as established by the Australian Bureau of Statistics Act 1975 and the Census and Statistics Act 1905.

The ABS does not mandate ethics approval for all PLIDA projects. Ethics approval is required by some data custodians for the use of specific datasets and may also be required by universities and other institutions with project oversight.

## Data Availability

PLIDA data are available to approved projects by application to the Australian Bureau of Statistics.
